# Grabody B, an IGF1R‐targeting Molecular Shuttle, Sustains Brain Penetration of Therapeutic Antibody in Aged Mice by Multiple, Novel Transcytosis Pathways

**DOI:** 10.1002/alz70855_103122

**Published:** 2025-12-24

**Authors:** Sungwon An, Dongin Kim, Miran Yoo, Hyesu Yun, Sumin Hyeon, Seung‐Hwan Kwon, Dongwhan Kim, Bruce Kim, Hakju Kwon, Do‐Geun Kim, Sang Hoon Lee

**Affiliations:** ^1^ ABL Bio Inc, Seongnam‐si, Gyeonggi‐do, Korea, Republic of (South); ^2^ Korean Brain Research Institute, Daegu, Dong‐gu, Korea, Republic of (South)

## Abstract

**Background:**

Molecular shuttles have been actively developed to overcome the limited delivery of therapeutics due to the blood brain barrier (BBB) by targeting receptors including transferrin receptor (TfR) and insulin‐like growth factor receptor 1 (IGF1R), and CD98 heavy chain (CD98hc) expressed on the brain endothelial cells (BECs). However, how the shuttles are trafficked into the BECs are yet to be identified in detail.

**Methods:**

Grabody B is the novel molecular shuttle as an anti‐IGF1R antibody. Grabody B‐fused bispecific antibodies (GB BsAbs) were proven to have increased brain exposure and efficacy compared to monoclonal antibodies (mAbs). Grabody B‐fused BsAbs penetrated into deeper cortical areas and recognized targets highly in cleared mouse brains. In contrast, mAbs were localized mostly near brain surfaces and close to ventricles, and perivascular areas of penetrating arteries.

**Results:**

To elucidate the underlying mechanisms of trafficking, we monitored Grabody B‐mediated endocytosis in human BECs. GB BsAbs rapidly internalized within 30 seconds, localized closely with filamentous actin (F‐actin), and even induced the reorganization of F‐actin with various morphologies. In contrast, TfR BsAbs internalized more slowly forming only dotted patterns. TfR is well known to utilize Clathrin, however, GB BsAbs tend to use both Clathrin and Caveolin. The fast internalization rate and close relation with F‐actin of Grabody B led us to investigate whether GB BsAbs utilize Fast‐endophilin‐mediated endocytosis (FEME). GB BsAbs strongly colocalized with endophilin A2, an essential component of FEME, in both ECs and mouse brain sections. However, TfR BsAb signals barely colocalized with endophilin A2. TfR BsAbs appear to follow the early endosomes, as shown by strong colocalization with Rab5 contrary to little or no GB BsAb signals colocalized with Rab5. According to recent literature, aged vessels were shown to express less Clathrin‐related machinery, and TfR BsAbs have lower brain penetration in older mice compared to the young mice. Interestingly, GB BsAbs exhibited comparable brain penetration between 2‐months‐old and 18‐month‐old animals.

**Conclusion:**

We confirm that Grabody B improves brain penetration of therapeutics with avoidance of the BCSFB‐related routes. Grabody B utilizes multiple transcytosis pathways including Clathrin, caveolin, and FEME, which enables sustained brain penetration in aged rodents.

